# The Effect of Periodization on Training Program Adherence

**DOI:** 10.3390/ijerph182412973

**Published:** 2021-12-09

**Authors:** Vicente Javier Clemente-Suárez, Domingo Jesús Ramos-Campo, José Francisco Tornero-Aguilera, Jose A. Parraca, Nuno Batalha

**Affiliations:** 1 Faculty of Sports Sciences, Universidad Europea de Madrid, 28670 Madrid, Spain; vctxente@yahoo.es (V.J.C.-S.); josefrancisco.tornero@universidadeuropea.es (J.F.T.-A.); 2 Grupo de Investigación en Cultura, Educación y Sociedad, Universidad de la Costa, Barranquilla 080002, Colombia; 3 Departamento de Salud y Rendimiento, Universidad Politécnica de Madrid, 28040 Madrid, Spain; domingojesusramos@gmail.com; 4 Departamento de Desporto e Saúde, Escola de Saúde e Desenvolvimento Humano, Universidade de Évora, 7005-869 Évora, Portugal; nmpba@uevora.pt; 5 Comprehensive Health Research Centre (CHRC), Universidade de Évora, 7005-869 Évora, Portugal

**Keywords:** adherence, endurance, periodization, triathlon

## Abstract

The present research aimed to study the effect of three different training periodization (traditional, reverse, and free training) on the aerobic performance, motivation, and adherence of physically active athletes. We analysed the adherence to three different periodization training programs: traditional, reverse, and free training periodization on the adherence of amateur triathletes. For this aim, the individual adherence, motivation, and aerobic performance time and heart rate (in a 1000 m running test) were evaluated before and after the completion of the three different 8-week periodization programs. The level of adherence to the reverse periodization was significantly higher than in traditional and free training. The number of dropouts in reverse and traditional periodization was similar but lower than in the free training. Finally, neither of the periodization programs improved aerobic performance and reverse training periodization decreased heart rate of participants in a 1000 m running test.

## 1. Introduction

The World Health Organization (WHO) defines adherence as “the extent to which a person’s behaviour—taking medication, following a diet, and/or executing lifestyle changes, corresponds with agreed recommendations from a health care provider.” In the sports field, adherence to training is the degree of fulfilment by athletes to tasks of all kinds that the training entails.

Ideally, due to its intrinsic characteristics, sport and competitive context make it ideal to promote sports commitment and, as long as the athlete achieves adequate motivation, adherence to physical-sports practice [1]. Yet, it is common to see inappropriate adherence, either by default or by excess, which can be decisive. Inappropriate adherence can occur with respect to regular training sessions or in relation to various complementary measures (preventive physiotherapy, pharmacological treatment, psychological training [2]). Authors suggest that over 50% of subjects who enrol in a training program will drop out after the first six months [3,4], with the main reasons relating to failure, lack of improvement, or changes in motivation [5]. Thus, motivation is a determining and key factor, and is the most repeated in the scientific literature according to recent systematic reviews [6,7], along with the figure of the coach on the behaviour and the motivation of his or her athletes [8]. Lack of motivation will lead to a failure of program adherence, and this is a big problem to exercise periodization, since it is based on chronic changes in the athlete organism, produced by an accumulation of the acute effects produced by different workloads. Thus, compromising the accumulative effect of workloads will diminish the chronic effects of the periodization plan, leading to failure and lack of improvements [9].

Likewise, the different models of periodization seem to have an influence on the motivational and adherence state of athletes. Colquhoun et al. 2017 [10] compared the effect of traditional and flexible daily undulating periodization on athlete’s adherence to the program. Both types of periodization are based on a nonlinear undulating distribution of the volume and intensity of workloads [11,12]. However, flexible daily undulating periodization allows the lifter to choose the order of the training sessions within a given time frame. Results showed that allowing participants to choose the order of workloads increased adherence to the program. McNamara and Stearne 2010 [13] also conducted a similar study comparing the same two types of periodization, while also giving the subjects in the flexible type of undulating periodization the ability to choose what repetition scheme they used each session. The ability of athlete’s to influence their workouts showed beneficial effects over adherence and athlete’s performance gains [13]. Yet, there are few studies regarding reverse periodization, that is, where athletes start their preparation with high-intensity and low-volume training, while gradually decreasing intensity and increasing volume or, depending on the sport, maintaining intensity, and increasing volume during the following training periods [14,15,16]; a training paradigm opposite to the traditional training periodization based on developing high-volume and low-intensity training during the first periods of the periodization, with progressive increases in training intensity and simultaneous decreases in training volumes of the consecutive periods [17,18].

In line reverse periodization, based on intensity training, the program training session is based on high intensity interval training. These training models have shown higher adherence and motivation than traditional continuous training, a fact related to the diversity of intensities of high intensity interval training, the lower duration of this training, and a lower monotony during the training sessions [19,20]

The extent to which athletes adhere to sport development programs is an important issue for applied sport scientists since it can help to improve the development of adherence to training programs and methodologies. Examining different training periodization methodologies, such as traditional, reverse, and free training periodization, it was found that one of the main modifiable and influencing factors during the training process is motivation, which is essential. Despite the training being prescribed in an athletic, recreational, or clinical situation, motivation and adherence are always major variables in determining the program’s effectiveness [10]. Thus, the present research aimed to study the effect of three different training periodization (traditional, reverse, and free training) in the aerobic performance, motivation, and adherence of physically active athletes. We hypothesized that reverse training periodization would present higher aerobic performance, motivation, and adherence than traditional and free periodization models, according to the results presented by previous studies.

## 2. Materials and Methods

### 2.1. Participants

A total of 30 physically active athletes were analysed (20 males: 26.8 ± 4.3 years; 174.6 ± 4.6 cm; 71.3 ± 5.7 kg; and 10 females: 24.3 ± 3.2 years; 165.2 ± 5.2 cm; 57.2 ± 4.5 kg) participated in the present research. They were randomly divided into three training groups: reverse training periodization (RTP, *n*:10), traditional training periodization (TTP, *n*:10), and free training (FT, *n*:10). The study design and the procedures employed were in accordance with ethical standards and the Declaration of Helsinki, being approved by the university ethics committee (CIPI/002/17). Each participant was fully informed of the risks associated with the study, and they gave written informed consent before the start of the study.

### 2.2. Procedures and Design

**Figure 1:**
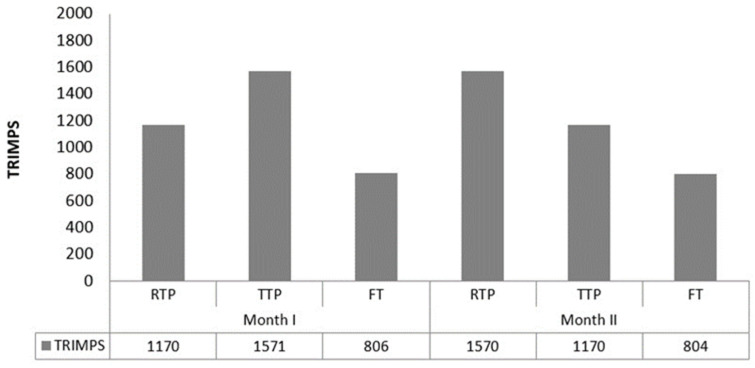
Distribution of Training Impulse (TRIMP) of each experimental group in the first and second month of training.

In order to reach the study aim, participants were divided in three groups each one conducting a different training periodization (reverse, traditional, free) over 8 weeks. Before and after the 8 weeks the performance, adherence, and motivation of participants were evaluated. The traditional periodization model is based in the sequentialization of volume to intensity during the periodization. While reverse training periodization are based in an opposed paradigm, firstly training intensity and then volume [21]. Then, the RTP group started with an intensity-based training the first 4 weeks of the program and finished with volume-based training. The TTP performed in the opposite direction, and finally the FT trained without any control of the researchers. The training load of participants was analysed by the Training Impulse (TRIMP) method [22], quantifying the TRIMPS in the first and second month of training (Figure 1).

Running performance was measured by time at maximal effort over 2000 m, which is associated with the maximal aerobic speed measured in incremental tests conducted in a laboratory [15]. After a 10-min aerobic standardized warm up, participants were instructed to run 2000 m at maximal speed on a track surface (temperature 18.2 ± 1.4 °C; 61.3 ± 2.1% humidity). The final heart rate of participants was analysed by a Polar V800 (Polar, Kempele, Finland) following previous studies [23].

In order to analyse the motivations of participants with each training program, they were asked after finalization of the 8 weeks of training to identify their motivation with the training conducted using 0 (low motivation) to 10 (high motivation scale). To analyse the adherence of participants, the number of participants in each training group was registered before and after the 8 weeks of training.

### 2.3. Statistical Analysis

Data were analysed with the SPSS (IBM, NY, USA) for Windows statistical package (v.21.0). Firstly, descriptive statistics (mean and standard deviation) were calculated. Before using parametric tests, the assumption of normality and homoscedasticity were verified using the Kolmogorov-Smirnov test. A MANOVA with training groups as fixed factor was conducted to analyse differences in the two-moment analysis of every variable between the three groups. A dependent T test was used to analyse modification in pre- and post-training interventions in each group. For all procedures, a level of *p* ≤ 0.05 was selected to indicate statistical significance.

## 3. Results

Reverse training periodization produced a significant decrease in HR of participants, but traditional and free training maintained their HR after the 8-week training period. None of the training groups significantly modified their performance in the running test (Table 1). The basal HR presented significant differences between free training and reverse and traditional training groups. No differences were found in the 2000 m time between groups in the basal sample, as well as in the HR after the 8-weeks of training. Nevertheless, there were significant differences between the 2000 m time between free and reverse training groups after the 8-weeks training intervention (Table 2).

Reverse training periodization showed a significantly higher motivation with training (9.2 ± 1.1) than traditional (8.0 ± 0.8, *p*:0.033) and free training (7.4 ± 1.1 *p*:0.029). In addition, traditional periodization also showed a significantly higher motivation with training than free training (*p*:0.041). Regarding the adherence to the training programs, there were no significant differences between groups, since only the free training group lost one participant, while the reverse and traditional periodization groups maintained the same number of participants.

## 4. Discussion

To the best of our knowledge, this is the first study that analysed the effect of three types of periodization programs (reverse, traditional and free periodization) on adherence, motivation and cardiovascular and performance variables in physically active recreational athletes. The main findings of the present study were: (i) The three types of periodization maintained the 1000-m running performance; (ii) Reverse training periodization promoted significantly higher motivation than traditional periodization and free training. In addition, traditional periodization also showed significantly higher motivation with training than free training; and (iii) adherence to the training program was similar in the three types of training periodization.

It has previously been reported that athletes that followed a structured and programmed training program had an increase in motivation to the training than unstructured training [24]. Moreover, periodized training prevents negative mood states associated with burn out and reduces boredom associated with monotonous training [24]. In addition, motivation positively influences adherence to the training, and both have been shown to be essential to generate a health behaviour and to have an active lifestyle [25]. There are some reasons that can affect motivation during a training program. First, athletes that performed periodized training increased their fitness and performance, provoked more body composition changes and obtained more physiological adaptations than athletes who followed a free and unstructured training program [26,27]. In this way, a relationship between motivation and an increase in athletic performance has been shown [28]. However, the results of the present study only found differences in the cardiovascular adaptations among the three training programs. The duration of the program (only 8 weeks) can explain the lack of differences in running performance. Second, reverse periodization is a new type of model and triathletes did not perform this type of training program previously. This fact can affect the self-expanding construct in the participants. Interestingly, self-expanding activities are characterized by novelty, excitement and interest or change [29], and positively influences motivation, improves health behaviours [30] and leads to increase effort and persistence in task [31], which can improve adherence to the program [24]. In this way, self-expansion occurs naturally but the type of training periodization could promote expansion effects due to the inclusion of short- and long-term goals, variety, and the promotion of continued progression of physiological factors and skill-acquisition [24]. Therefore, periodized methods and specifically reverse periodization have a strong potential to promote motivation to a greater degree than non-periodized programs. Finally, the characteristic high intensity training that is used in reverse training periodization, since the beginning of the training periodization, differs from other periodization models and could promote a higher hedonic response than monotonous continuous training [32]. This fact could contribute to increased motivation of athletes and probably the adherence to this program in long-term sport interventions.

Reverse periodization, unlike previous periodization models, begins the macrocycle with high-intensity and low-volume training, while gradually decreasing intensity and increasing volume or, depending on the sport, maintaining intensity and increasing volume during the following training periods [15]. The main adaptations associated with this type of periodization (reverse periodization) that provoke an increase of athletic performance are closely related with the application of high intensity training [16] (e.g., increase sympathetic modulation to achieve different physiological adaptations related with aerobic performance such as the increase in GLUT4 concentration, the optimization of the muscle buffering capacity, the increment of the maximal glucose transport activity in skeletal muscle or the enhancement of the glycogen content [33,34,35,36]). Moreover, compared to moderate continuous training (commonly used in traditional periodization) high intensity training induces similar-to-greater improvements in fitness and cardiovascular function but in a shorter amount of time [34]. In addition, previous studies reported [37,38] that high intensity training is more enjoyable, motivational, and promotes more adherence to the program than aerobic low/moderate continuous training. In the present study, performance changes and training load were similar in traditional or reverse periodization. However, taking into consideration the effect of the success of the training program across lifespan, the motivational results point to reverse periodization as a promising periodization for promoting exercise enjoyment, motivation, and adherence in physically active young adults.

Many guidelines for physical activity and exercise published by international associations [39] have recommended an increase in cardiovascular fitness, along with maximal strength and the improvement of body composition, to provide overall health benefits in the young [40] and elderly population [41], as well as for the improvement of quality of life in patients with different pathologies [42]. Coaches and physical educators need to take into consideration affective and motivation responses to training programs to obtain successful adherence and fulfil the recommendations made for improved quality of life. In this way, and from a practical application point of view, if coaches want to obtain greater motivation in participants who perform their training program, they may choose to use a reverse periodization model rather than traditional periodization or free training. On the other hand, we acknowledge some limitations in the present study. In this way, the main limitation of the present study was the small sample size analysed, which limited the generalization of the result. Furthermore, we could not perform a randomized controlled crossover because of the impossibility of maintaining participants’ participation over a long time period due to their club training and competition commitments. Additionally, analysing the psychological profile of participants would improve knowledge of the motivation response of recreational athletes. Finally, future studies should compare other types of periodization and identify the mechanism involved in motivation and adherence to exercise. Furthermore, previous studies found that the providers’ excess weight may negatively affect patients’ perceptions of their credibility, level of trust, and inclination to follow medical advice [43]. Moreover, coaches that applied an autocratic coaching style negatively influence related psychological factors or affective factors affecting neuroendocrine response patterns, self-confidence, and motivational climate [44]. Thus, whether coaches’ body weight and their coaching style affects athletes’ motivation and adherence needs to be analysed.

## 5. Conclusions

The level of adherence to the reverse periodization was significantly higher than in traditional and free training. The number of dropouts in reverse and traditional periodization was similar but lower than in the free training. Finally, neither of the periodization programs improved aerobic performance, and reverse training periodization decreased heart rate of participants in a 1000 m running test.

## Figures and Tables

**Table 1:** Results of heart rate and 1000 m time before and after each training program.

	Reverse				Traditional				Free			
	Pre	Post	T	*p*	Pre	Post	T	*p*	Pre	Post	T	*p*
Heart rate (bpm)	190.4 ± 9.4	184.2 ± 11.0	−3.7	0.004	182.4 ± 7.4	184.3 ± 6.4	−1.0	0.358	185.0 ± 12.2	188.3 ± 13.2	0.5	0.652
1000 m time (s)	242.5 ± 26.6	237.8 ± 25.4	1.2	0.278	240.1 ± 28.3	236.8 ± 25.4	2.0	0.064	266.0 ± 45.2	268.5 ± 38.5	−5.6	0.594

**Table 2:** Differences in heart rate and 1000 m time between experimental groups before and after the training programs.

Evaluation Moment	Variable	Group	Group	*p*	95% Confidence Interval	
					Lower	Upper
Pre	Heart rate (bpm)	Reverse	Traditional	1.000	−3.7037	5.9233
			Free	0.004	2.1340	13.0532
		Traditional	Reverse	1.000	−5.9233	3.7037
			Free	0.012	1.2040	11.7636
		Free	Reverse	0.004	−13.0532	−2.1340
			Traditional	0.012	−11.7636	−1.2040
	1000 m time (s)	Reverse	Traditional	1.000	−35.1329	41.7880
			Free	0.256	−74.1885	13.0567
		Traditional	Reverse	1.000	−41.7880	35.1329
			Free	0.151	−76.0796	8.2926
		Free	Reverse	0.256	−13.0567	74.1885
			Traditional	0.151	−8.2926	76.0796
Post	Heart rate (bpm)	Reverse	Traditional	0.349	−21.0515	4.6629
			Free	1.000	−18.7081	10.4577
		Traditional	Reverse	0.349	−4.6629	21.0515
			Free	1.000	−10.0336	18.1717
		Free	Reverse	1.000	−10.4577	18.7081
			Traditional	1.000	−18.1717	10.0336
	1000 m time (s)	Reverse	Traditional	1.000	−32.7898	40.6065
			Free	0.098	−78.3655	4.8821
		Traditional	Reverse	1.000	−40.6065	32.7898
			Free	0.047	−80.9032	−0.3970
		Free	Reverse	0.098	−4.8821	78.3655
			Traditional	0.047	0.3970	80.9032

## Data Availability

All data are in the manuscript.
